# Potential of mucoadhesive chitosan glutamate microparticles as microbicide carriers – antiherpes activity and penetration behavior across the human vaginal epithelium

**DOI:** 10.1080/10717544.2021.1992037

**Published:** 2021-10-20

**Authors:** Emilia Szymańska, Małgorzata Krzyżowska, Krzysztof Cal, Barbara Mikolaszek, Jakub Tomaszewski, Sławomir Wołczyński, Katarzyna Winnicka

**Affiliations:** a Department of Pharmaceutical Technology, Medical University of Białystok, Bialystok, Poland; b Department of Rheumatology and Inflammation Research, University of Gothenburg, Gothenburg, Sweden; c Military Institute of Hygiene and Epidemiology, Warsaw, Poland; d Department of Pharmaceutical Technology, Medical University of Gdańsk, Gdańsk, Poland; e Private Obstetric and Gynecological Clinic, Tomaszewski Medical Centre, Białystok, Poland; f Department of Reproduction and Gynecological Endocrinology, Medical University of Białystok, Bialystok, Poland

**Keywords:** Chitosan glutamate, microbicide vaginal carrier, microparticles, mucoretention, ex vivo penetration studies, human vaginal epithelium, HSV-2, antiviral activity

## Abstract

Chitosan glutamate (gCS) spray-dried microparticles appear promising carriers to overcome challenges associated with vaginal microbicide delivery. This study aimed at elucidating the penetration and mucoadhesive behavior of developed gCS multiunit carriers with zidovudine (ZVD) as a model antiretroviral agent in contact with excised human vaginal epithelium followed with an examination of *in vitro* antiherpes activity in immortal human keratinocytes HaCaT and human vaginal epithelial cells VK2-E6/E7. Both ZVD dispersion and placebo microparticles served as controls. Microparticles displayed feasible (comparable to commercial vaginal product) mucoadhesive and mucoretention characteristics to isolated human vaginal tissue. *Ex vivo* penetration studies revealed that gCS increased the accumulation of active agent in the vaginal epithelium but surprisingly did not facilitate its penetration across human tissue. Finally, the obtained antiviral results demonstrated the potential of gCS as an antiherpes adjunctive, whose mode of action was related to blocking viral attachment.

## 1. Introduction

Sexually transmitted diseases (STDs), including bacterial, viral, and parasites infections are relevant and still existing public health issues worldwide (Shannon & Klausner, 2018; Seña et al., 2020; World Health Organization, 2021). In recent years, the prevalence of genital herpes caused by herpes simplex virus type 1 or 2 (HSV-1/2) has gradually increased, and resistance to conventional antiviral drugs (e.g. acyclovir) has been reported (McQuillan et al., 2018). HSV increases the risk of HIV acquisition by forming breaches in the genital epithelium and creating a state of chronic inflammation. It should be noted that HSV is able to reactivate periodically, allowing the transmission to occur, even in the absence of clinical symptoms (Sauerbrei, 2016). At present, the conventional treatment (by oral acyclovir and its analogues) does not cure but basically help to reduce the duration and severity of recurrent genital herpes. Therefore, there is a need to develop novel strategies aiming at preventing either new infection or frequent recurrence of HSV episodes.

Among goals and priority actions established by the WHO Global Health Sector Strategy on Sexually Transmitted Infections (2016–2021), vaginal microbicides present a promising prophylactic approach against STDs, including genital herpes (World Health Organization, 2021). Microbicides refer to topically (vaginal or rectal) applied antimicrobial or antiviral agents intended to prevent infections (Karim & Karim, 2007). Several classes of microbicides have been proposed as HSV prophylactic strategy (AVAC, 2021; Clinical Trials, 2021; Cordis, 2021), but effective and safe products are still not available. Although efforts have been made toward finding and testing proper active microbicide agents, currently, more focus has been made on the development of novel delivery systems which ideally would provide the patient with a convenient vaginal application and simultaneously preserve or even enhance the protecting microbicide effect (Mesquita et al., 2019).

Chitosan is a natural copolymer derived from chitin which consists of glucosamine and N-acetylglucosamine units. With regard to its cationic nature, chitosan binds to mucosal surfaces and thus has been extensively studied for a number of biomedical and pharmaceutical applications (Ahmed & Aljaeid, 2016; Ahsan et al., 2018; Potaś et al., 2020). We have previously shown that chitosan could be successfully used in the technology of carriers for vaginal candidiasis treatment (Szymańska et al., 2014). This capability of increasing the pharmacological action of an antimicrobial agent provides the opportunity for combination therapy in which chitosan acts as the effective excipient and simultaneously takes an active part in the treatment process. Apart from antifungal activity, chitosan has gained attention as an antiviral adjunctive directly impacting virus infectivity (Russo et al., 2014; Loutfy et al., 2020; He et al., 2021; Safarzadeh et al., 2021). In our previous studies, we observed that upon contact with vaginal fluid, chitosan creates a swellable hydrogel matrix which may serve as a barrier and additionally support mucosal tissue from the risk of infection (Szymanska et al., 2019).

Vaginal administration (including microbicides) is considered beneficial as it allows administration of lower drug dose, maintains its steady state levels and is not affected by gastrointestinal disturbances when compared to the oral route (Swarbrick, 2007). However, effective treatment with conventional vaginal preparations (creams, globules) containing antimicrobial drugs is often inconvenient for patients, which results in low product acceptability. What is more, until now, there are no vaginal microbicide formulations available on the pharmaceutical market.

We have recently demonstrated a feasible potential of spray-dried microparticles composed of water-soluble chitosan derivative – chitosan glutamate (gCS) as a microbicide delivery system for vaginal application (Szymanska et al., 2019). By applying an experimental design approach, the technology of spray-drying gCS microparticles was optimized, and the obtained multiunit carriers were characterized by favorable physical and biopharmaceutical properties.

This study aims at investigating the biological potential of developed gCS multiunit carriers loaded with zidovudine (ZVD) – model antiretroviral agent belonging to a class of reverse transcriptase inhibitors. Apart from mucoadhesive potential, the precise goal was to examine the effect of gCS microparticles against HSV-2 in two relevant cell lines: immortal human keratinocytes HaCaT and human vaginal epithelial cells VK2-E6/E7. Taking into consideration that chitosan particles are considered to improve the bioavailability of active agents by enhancing their transport through the epithelial cells (Caramella et al., 2010), particular effort was made toward evaluation *ex vivo* penetration and retention behavior of a model microbicide agent encapsulated in gCS multiunit formulations across the human vaginal epithelium.

## 2. Materials and methods

### 2.1. Materials

Highly purified medical grade gCS was purchased from Heppe Medical Chitosan (Haale, Germany). Molecular weight (Mn 101 kDa) and average molecular weight (Mw 247 kDa) were determined with PVP-calibration on Agilent 1260 Infinity GPC/SEC at 35 °C with a refractive index detector (Agilent Technologies, Santa Clara, CA). The deacetylation degree (80.2%) was determined by titration method according to Czechowska-Biskup et al. (2012), whereas the viscosity of 1% (w/w) aqueous solution (30 mPas) was measured with rotational viscometer Viscotester 6 Plus ThermoHaake (Karlsruhe, Germany) at ambient temperature. The protein and sulfated ashes content was below 0.5% and 1%, respectively (according to the manufacturer).

Zidovudine in crystalline form (batch no. ZD1900515) was obtained from Hetero Labs Limited (Hyderabad, India). Methanol (HPLC grade) was obtained from Merck (Darmstadt, Germany), sodium azide was from Sigma-Aldrich (Darmstadt, Germany), whereas hydroxyethylcellulose (HEC) (with viscosity of 1% (w/w) aqueous solution of approximately 40 mPas at 25 °C) was purchased from Fluka (Buchs, Switzerland). Water for HPLC was distilled and passed through a reverse osmosis system Milli-Q Reagent Water System (Billerica, MA). Simulant vaginal fluid (SVF) pH 4.2 for mucoadhesive and penetration tests was obtained according to Owen & Katz (1999) with the following composition (g): sodium chloride, 3.51; lactic acid, 2.00; potassium hydroxide, 1.40; calcium hydroxide, 0.22; acetic acid, 1.00; urea, 0.4; glycerol, 0.16; and glucose, 5.0 and water up to 1000 mL. Other chemicals were purchased from Chempur (Piekary Śląskie, Poland).

Dulbecco’s modified Eagle medium (DMEM), defined keratinocyte serum-free medium (Ker-SFM), minimum essential medium with alpha modification (α-MEM), fetal calf serum (FCS), phosphate buffer saline (PBS), human recombinant epidermal growth factor (EGF), bovine pituitary extract, penicillin, and streptomycin were purchased from Thermo Fisher Scientific (Poznań, Poland). HSV-2 strain 333 isolate received from the University of Gothenburg (Gothenburg, Sweden) was propagated in Vero cells. Immortal human keratinocytes cell line HaCaT was from Cell Line Service CLS GmbH (Eppelheim, Germany), whereas human vaginal epithelial cells VK2-E6/E7 (CRL 2616) were purchased from American Type Culture Collection (Manassas, VA). A plasmid vector pCR 2.1 containing envelope glycoprotein (gB) gene fragment was constructed and purified by the Institute of Biochemistry and Biophysics Polish Academy of Sciences (Warsaw, Poland).

### 2.2. Human vaginal epithelium

Local Bioethics Committee (Medical University of Białystok) revised and approved the study protocol using human tissue under the no. R-I-002/462/2018. Freshly excised human vaginal epithelium was obtained from the Private Clinic of Obstetrics and Gynecology (Bialystok, Poland) from premenopausal women undergoing gynecological plastic surgery. Apart from age, no information concerning the patient’s identity was disclosed. Directly after the surgery, tissue specimens were preserved in the isotonic saline solution and kept at −20 °C for no longer than 60 days. In prior experiments, tissue was thawed at room temperature, cut into pieces (weight 300–350 mg) and microscopically checked for tissue integrity.

### 2.3. Microparticles preparation

Placebo (P) and two drug-loaded formulations MB1 and MB2 differed in gCS:ZVD ratio 2:1 and 5:1 (w/w), respectively, were obtained by using a Bűchi Mini Spray Dryer B-290 (Flawil, Switzerland) according to the method previously described by our group (Szymanska et al., 2019). Briefly, 3% (w/w) gCS aqueous dispersion was mixed with a water–ethanol drug solution under continuous stirring. Based on previously established studies, spray drying was performed at 120 °C in an opened loop configuration at a spray rate of 1.8 mL/min with aspirator blower capacity (95%) and pressure (40 mm Hg). The production yield was 75%.

### 2.4. Microparticles characterization

Microparticles were characterized in terms of moisture, ZVD content, and encapsulation efficiency evaluated using HPLC method after previously described extraction procedure (Szymanska et al., 2019) and volume particles size distribution (examined using light diffraction analyzer Mastersizer 3000 equipped with HydroEV probe; Malvern Instruments, Malvern, UK).

The morphology of microparticles was examined by a scanning electron microscope (SEM, Phenom Pro Generation 5, Thermo Fisher, Eindhoven, Netherlands) by in-line detection mode at 5 kV or 10 kV with a BSD detector. Before the microscopic observations, approximately 0.5 mg of powder was spitted on conductive carbon adhesive tabs and coated with a thin layer of gold in an ion sputtering device (4.9 nm). The obtained micrographs were evaluated for particle size and shape and tendency to create agglomerates. The surface of the microparticles was assessed, with a focus on the presence of cracks in the shells or ZVD crystals.

Viscosity measurements were conducted at ambient conditions with rotational viscometer Viscotester 6 Plus ThermoHaake (Thermo Scientific, Braunschweig, Germany) equipped with a rotor TL 6 (for formulation MC1) and TL 7 (for formulation MC2 and P). Before tests, each microparticles sample (whose amount corresponded to drug dose 10 mg/mL) was placed in a vial containing SVF pH 4.2 and shook thoroughly for 2 h at ambient temperature until complete dispersion of the sample. Tests were carried out with a rotational speed of 20/min 10 min after placing the preparation’s sample (10 mL) into the measuring tube. The values of dynamic viscosities were noted after 30 s. The results were shown as the average of three independent tests.

### 2.5. Mucoadhesive measurements

The *ex vivo* mucoadhesive behavior of microparticles in contact with human vaginal epithelium was assessed with texture analyzer TA.XT. Plus (Stable Microsystems, Godalming, UK) equipped with an A/MUC measuring system at 37 ± 2 °C as previously described (Szymańska et al., 2018). A sample of placebo or MB microparticles (100 mg) was adhered to the platform A/MUC with bioadhesive tape and wetted with 100 µL of SVF (pH 4.2), whereas the tissue sample (surface area 0.8 cm^2^) was fixed with cyanoacrylate glue to the upper tester probe. Afterwards, the probe was lowered onto the surface of microparticles with a constant speed of 0.5 mm/s. After keeping a contact time for 100 s under an initial contact force of 0.5 N, the planes were separated at a constant rate of 0.1 mm/s. The maximum detachment force was recorded directly from Texture Exponent 32 software, and the work of mucoadhesion was measured from the area under the force vs. distance curve. Cellulose paper was applied as a negative control. In contrast, a commercially available mucoadhesive vaginal product with lubricating properties Replens MD^TM^ composed of polycarbophil, carbomer homopolymer type B, hydrogenated palm oil glyceride, glycerin, mineral oil, sorbic acid, sodium hydroxide, and purified water (Lil Drug Store Products, Cedar Rapids, IA, US) was used as a positive control. Each experiment was performed five times.

### 2.6. Mucoretention behavior upon dilution with simulant vaginal fluid

To examine mucoretention behavior of microparticles formulations upon dilution with SVF (pH 4.2), the measurements were carried out using human vaginal epithelium attached to a self-constructed thermostated inclined steel plate at 36 ± 2 °C. The kinetic detachment was determined from the delay in the sample slipping and the overall detachment from the mucosal tissue (Balasch-Risueño & López, 2019). Before analysis, MB1 and MB2 or placebo formulation in the amount corresponding to MB2 was placed in a vial containing SVF (pH 4.2) and shook thoroughly until complete sample dispersion. The average SVF volume of 3 mL was chosen for studies according to data that daily production of vaginal fluid is 2–6 mL per day (Godley, 1985; Bernkop-Schnürch & Hornof, 2003) and the final ZVD concentration in microparticles’ dispersion was 10 mg/mL. Water and commercially available mucoadhesive vaginal product (diluted in a comparable manner as microparticles MB2) were used as a negative and positive control. A diluted sample (1 mL) was gently spread on the human vaginal tissue (area 1 cm^2^) and fixed with cyanoacrylate glue to a horizontally positioned plate and left for 5 min. To start the run, the plane was set at a 45° inclination which simulated changing body positions. The amount of sample detached from the tissue was weighted (Radwag XA 60/220, Radom, Poland) after 30 s, 1 min, 3, 5, 10, and 30 min. Each value was compared with the initial weight of the applied sample to build a mucoretention vs. time plot. The average of three independent registered weights at each time interval was measured. The mucoretention expressed as a percentage of the sample adhered to mucosal tissue was calculated through the following formula:

$$\text{Mucoretention}=(P_{0}-P_{\text{t}})/P_{0}\times 100$$

where *P*_0_ is the initial sample weight applied on the tissue plane and *P*_t_ is the weight of the sample detached from the tissue plane at each time interval.

### 2.7. Penetration studies

Penetration studies were conducted in a flow-through cell system equipped with thermostated Teflon Bronaugh diffusion chambers in accordance with draft guideline on quality and equivalence of topical products published by the European Medicine Agency (European Medicines Agency, 2021). In brief, the human vaginal epithelium was sandwiched between the two compartments with the epithelial side facing the donor compartment and equilibrated with isotonic saline solution (pH 6.6) for 60 min. The diffusion area of the tissue was 0.81 cm^2^. To exclude the impact of drug release behavior on penetration characteristics, microparticles were dispersed in SVF prior to penetration testing. For this purpose, the proper amount of MP formulation (which amount corresponded to 5 mg ZVD dose) or pure ZVD was suspended in 0.5 g of SVF or SVF with the addition of 3% (w/w) HEC as a universal placebo gelling agent, respectively. The donor compartments of all chambers were protected with parafilm. The acceptor medium (isotonic saline solution with the addition of 0.005% sodium azide, pH 6.6) was then recirculated beneath the vaginal tissue at a constant rate of 30 mL/h. At predetermined time intervals (1, 2, 4, 6, 8, and 24 h), samples of acceptor fluid were withdrawn, filtered through cellulose acetate filters 0.2 µm and analyzed directly for drug content using HPLC method. The samples were replaced with the same volume of acceptor solution to assure sink conditions. Permeation profile was formed by plotting the cumulative amount of ZVD permeated through the human vaginal epithelium per unit surface area (μg/cm^2^) vs. time. Permeability flux expressed as changes in concentration of active agent permeated to acceptor medium per unit area per unit time was calculated as follow:

Flux=Δc/Δt×V/A

where Δ*c*/Δ*t* represents changes in concentration of the active agent in the acceptor compartment per unit time (µg/mL × h), *V* is the volume of acceptor compartment (mL), and *A* represents exposed surface area (cm^2^).

### 2.8. Assessments of drug retention and drug recovery

At the end of the studies, drug dispersion from the donor compartment was carefully aspirated to a flat-bottomed flask, washed with SVF (pH 4.2) until complete removal of drug from the top of the tissue and incubated in a water bath (1 h, 150 rpm, 30 °C). After centrifugation (4000 rpm, 15 min), the aspirate was filtered through cellulose acetate membrane filters 0.2 µm, diluted with mobile phase, and analyzed for drug content. The tissue was cut into pieces, immersed in methanol (10 mL), homogenized and incubated for 5 h in a water bath (30 °C, 150 rpm). After having filtered through nylon membrane filters 0.2 µm, the extract was analyzed for the amount of drug retained in the human vaginal epithelium using the HPLC method.

### 2.9. Solubility measurements

The equilibrium solubility of ZVD was measured at 37 ± 0.5 °C in SVF (pH 4.2) and isotonic saline solution. For this purpose, an excess of drug powder was added to the respective media and incubated in a water bath 24 h to reach equilibrium (37 °C and 150 rpm). Afterwards, samples were centrifuged (4000 rpm for 15 min) to remove the undissolved drug, filtered through a cellulose membrane filter (0.2 µm) and determined by HPLC system. All measurements were done in triplicate, and the average drug solubility was reported in milligrams per milliliter along with standard deviation.

### 2.10. Analytical method

Drug concentrations in acceptor fluid, extracts and aspirate from donor compartments were determined by reverse-phase high-pressure liquid chromatography (HPLC Agilent Technologies 1200, Waldbronn, Germany) according to the method (Dunge et al., 2005) with modifications. The separation was achieved by isocratic elution using Zorbax Eclipse C18-BDS column (150 × 4.6 μm, 5 μm) at 30 °C with a flow rate of 1 mL/min. The mobile phase composed of methanol and acetate buffer, pH 4.5 23:77 (v/v). HPLC validation method is summarized in Table 1. The presence of gCS, SVF (pH 4.2) or acceptor medium did not interfere with drug detection.

**Table 1.** RP-HPLC validation method summary.

Parameters	Value
Linearity (µg/mL)	0.5–20 µg/mL
Regression equation	60.856*x* + 0.6792
Regression coefficient (*R*^2^)	0.9955
Intra-day precision (% RSD)	1.89
Inter-day precision (% RSD)	1.94
Limit of detection LOD (µg/mL)	0.11
Limit of quantification LOQ (µg/mL)	0.34

### 2.11. Cell lines and viruses

HSV-2 strain 333 isolate was propagated and titrated in Vero cells, which were maintained in a complete culture medium consisting of α-MEM with 10% FBS, 100 U/mL penicillin, and 100 µg/mL streptomycin as previously described (European Medicines Agency, 2021). In contrast, human HaCaT keratinocytes were propagated in DMEM supplemented with 10% FCS, 10 U/mL penicillin, and 100 μg/mL streptomycin (Orlowski et al., 2014). The human epithelial vaginal VK2-E6/E7 cell line was maintained in Ker-SFM with the addition of 0.1 ng/mL human recombinant EGF, 0.05 mg/mL bovine pituitary extract, and additional calcium chloride 44.1 mg/L (at a final concentration of 0.4 mM).

### 2.12. HSV infection *in vitro*

*In vitro* activity of MB2 and corresponding placebo formulation toward HSV-2 infection in cultured HaCaT and VK2-E6/E7 cells was determined by attachment assay (Bagdonaite et al., 2015). For attachment tests, formulations diluted to 0.5 mg/mL in complete culture medium, mixed with HSV-2 (with a multiplicity of infection (MOI) 0.1) were added to 24-well plates containing pre-chilled (at 4 ± 0.5 °C for 1 h) HaCaT or VK2-E6/E7 cell monolayers (1 × 10^5^ cells per well). To allow viral attachment, plates were incubated for 1 h at 4.0 ± 0.5 °C. After removing the unattached formulations and unabsorbed HSV, cells were rinsed with PBS and overlaid with fresh culture medium, followed by further incubation at 37.0 ± 1 °C.

For penetration assays, HaCaT and VK2-E6/E7 cells were infected with HSV-2 (with a MOI 0.1) for 1 h at 4.0 ± 0.5 °C. Next, the unbound virus was washed away with cold PBS, and the cells were incubated with formulations diluted to 0.5 mg/mL in a complete culture medium for 1 h at 37 ± 1 °C. After this time, cells were rewashed and incubated for further 20 h at 37.0 ± 1 °C. Acyclovir with a concentration 100 mg/mL was used as a control. Studies were performed at least in triplicate.

### 2.13. HSV-2 titration

At 24 h post infection, total DNA was isolated from cell cultures using a DNA isolation kit (Eurx, Gdańsk, Poland). HSV-2 was detected using an HSV-2 probe labeled with carboxyfluorescein FAM in a real-time PCR instrument Stratagene MX4000 Real-Time qPCR System (Agilent Technologies, Santa Clara, CA) as described by Namvar et al. (2005). A plasmid vector pCR 2.1 containing envelope glycoprotein (gB) gene fragment was constructed and purified by the Institute of Biochemistry and Biophysics Polish Academy of Sciences (Warsaw, Poland). Standard curve analysis was based on Ct values and serial of 10-fold dilutions of the plasmid standard with an initial concentration of 2.62 × 10^6^ HSV-2 genome copy numbers per reaction. A standard curve was included in each PCR run. The amplification efficiency (*E*) was calculated from the standard curves, using the formula *E* = 10(–1/*a*) − 1, where *a* is the slope. Data are expressed as the HSV-2 copy number per ng of the total DNA in the culture.

### 2.14. Statistical analysis

Quantitative variables were expressed as the mean ± standard deviation. A statistical analysis was performed with the Statistica 12.0 software. The normality of results was checked using the Shapiro–Wilk test. The difference among the mean values of mucoadhesiveness, amount of drug permeated through vaginal tissue and antiviral efficacy were tested using the Kruskal–Wallis test with post hoc nonparametric Dunn’s test for multiple comparisons. Differences between groups were considered to be significant at *p* < .05.

## 3. Results and discussion

### 3.1. Microparticles characteristics

In present studies, the mucoadhesive and penetration behavior followed with *in vitro* efficacy toward HSV-2 of water-soluble chitosan derivative – gCS vaginal multiunit carriers with a model microbicide agent was investigated. Based on our previous studies, two formulations (MB1, MB2) obtained by the spray-drying technique varying in the mass ratio of polymer to the active substance (2:1 and 5:1, respectively) were selected for detailed analysis. Obtained formulations were characterized by a regular spherical shape, low moisture content, and high yield of ZVD encapsulation (Table 2) which point toward the good capacity of gCS to encapsulate the drug in the spray-drying process followed with the correct choice of process parameters.

**Table 2.** Characteristics of spray-dried drug-free (P) and ZVD-loaded microparticles (MB1–MB2) differed in chitosan glutamate to zidovudine mass ratio.

Formulation	Encapsulation efficiency^a^ (%)	Moisture Content (%)	Particle size distribution^b^ (µm)			Viscosity (mPas at 25 °C)^c^
			*D*_10_	*D*_50_	*D*_90_	
MB1	87.2 ± 0.8	5.2 ± 0.1	2.0	10.3	26.7	170
MB2	79.0 ± 1.2	5.0 ± 0.2	2.2	12.2	28.2	2420
P	–	4.3 ± 0.1	n.d.			2850

^a^ Percentage of the amount of active agent encapsulated in microparticles assessed by HPLC method (Agilent Technologies 1200) vs. theoretical drug content × 100.

^b^ *D*_90_ value describes cumulative percentage particle undersize values for 10%, 50%, and 90% of MB by volume; assessed by Mastersizer 3000 (Malvern Instruments, Malvern, UK); n.d. – not determined.

^c^ Measured with a rotational viscometer Viscotester 6 Plus ThermoHaake (Thermo Scientific, Braunschweig, Germany) upon microparticles dilution in SVF (pH 4.2) in the amount corresponded to drug dose 10 mg per mL.

**Figure 1.**
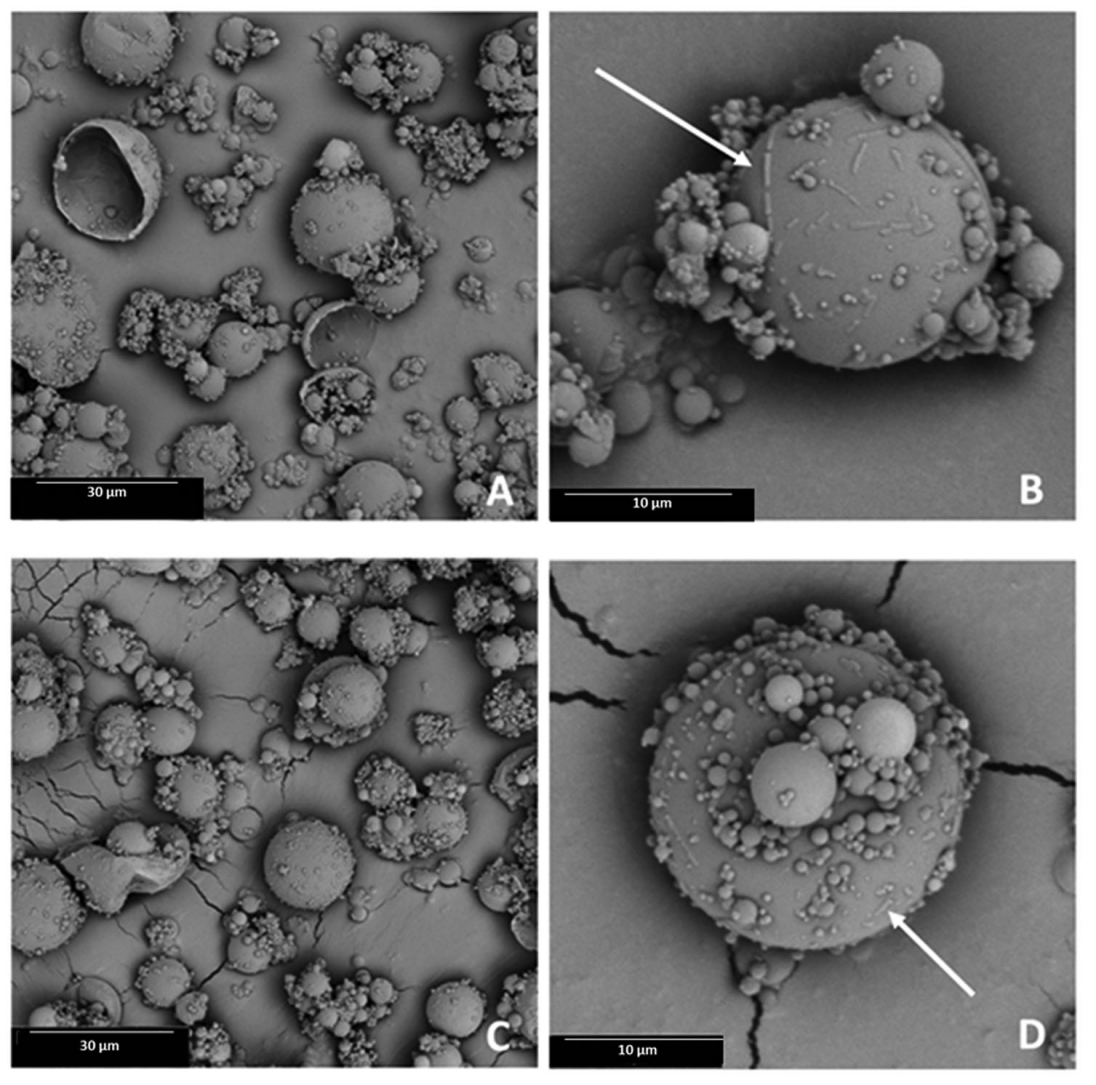
Scanning electron microscopy (SEM) images displaying microparticles MB1 (A, B) and MB2 (C, D) (original magnification: A, C, ×2000 and B, ×6000, D, ×8000).

Generally, microparticles did not differ in size, having a diameter below 30 µm (Table 2). The SEM analysis revealed the presence of substantial number of elongated drug crystals located superficially on the surface of MB1 and MB2 formulations (indicated by arrows on Figure 1). Both samples exhibited a tendency to create agglomerates, and a presence of smaller particles on the surface of larger ones was noted as a result of electrostatic interparticle forces. In Figure 1(A), some damaged hollow structures and cracks in the shells of polymer fragments can be distinguished. They indicate the higher amount of ZVD and polymer to drug ratio 2:1 led to the formation of more fragile particles during the spray-drying process, where a larger fraction of particles (above 20 µm) were susceptible to cracking.

### 3.2. Mucoadhesive behavior

**Figure 2.**
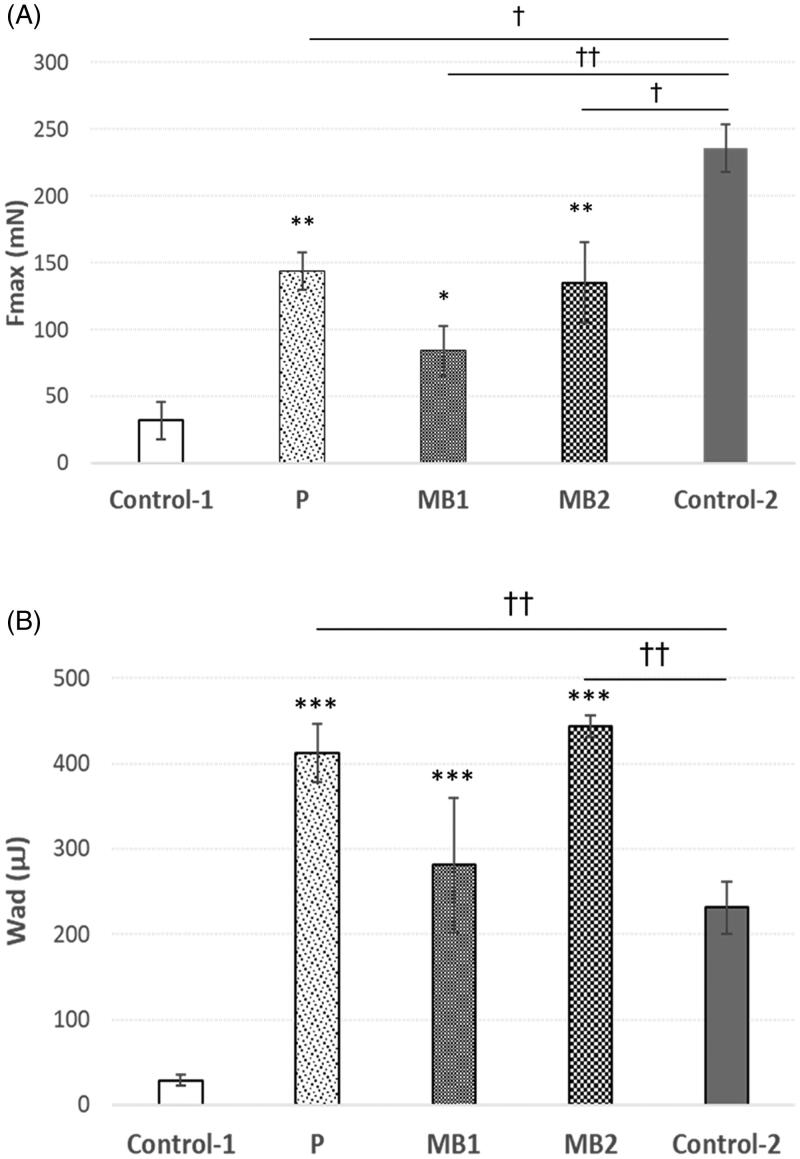
(A) Maximum force of detachment (*F*_max_) expressed in millinewtons (mN) and (B) work of mucoadhesion (*W*_ad_) expressed in microjoules (µJ) of microparticles MB1–MB2 and placebo formulation. Values are expressed as mean ± S.D., *n* = 5; *significant differences with *p* ≤ .05, ** with *p* ≤ .01 and *** with *p* ≤ .001 in comparison to control-1 (cellulose paper); ^†,††^significant differences with *p* ≤ .05 and *p* ≤ .01 in comparison to control-2 – commercially available mucoadhesive vaginal product, respectively.

*Ex vivo* tensometric measurements elaborated the strength required to separate tested material from the isolated human vaginal epithelium. Figure 2 shows the maximum detachment force (imitating the mechanical stress resulting from the rapid body movements disturbing the contact between formulation and mucosal tissue) and work of mucoadhesion (mimicking the overall capability to retain in the application site upon continuous changing body positions) of tested formulations MB1–MB2, placebo and controls.

All tested microparticles were capable of adhering to human mucosal tissue displaying mucoadhesive potential as compared to applied controls – cellulose paper and commercially available vaginal mucoadhesive product. After initial wetting with SVF (pH 4.2), the viscous film appeared on the microparticles’ surface, which favored further interpenetration of gCS and mucin chains. A certain influence of polymer to ZVD ratio on mucoadhesive behavior was noted. Formulation MB1 (with gCS:ZVD mass ratio 2:1) was found less adhesive, which points that presence of drug impairs interaction between mucoadhesive polymer and vaginal epithelium. Similar observations were made by Meng et al. (2011) in respect of vaginal microcarriers with tenofovir and in Dott et al. (2013) work on electrospun HPMC nanofibers with diphenhydramine where the addition of drug resulted in a reduction in formulations mucoadhesiveness. Among tested microparticles, placebo formulation displayed the greatest values of the force of mucoadhesion, though microparticles MB2 exhibited slightly higher values of work of mucoadhesion parameter. All formulations presented a similar manner of examined mucoadhesive properties (Figure 2(A,B)) except for commercial vaginal product, which exhibited the greatest value of maximum detachment force but the profoundly lower value of work of mucoadhesion as compared to results obtained for MB formulations. This observation may be rather attributed to the semi-solid state of the commercial mucoadhesive product and may suggest its higher susceptibility to body movements.

**Figure 3.**
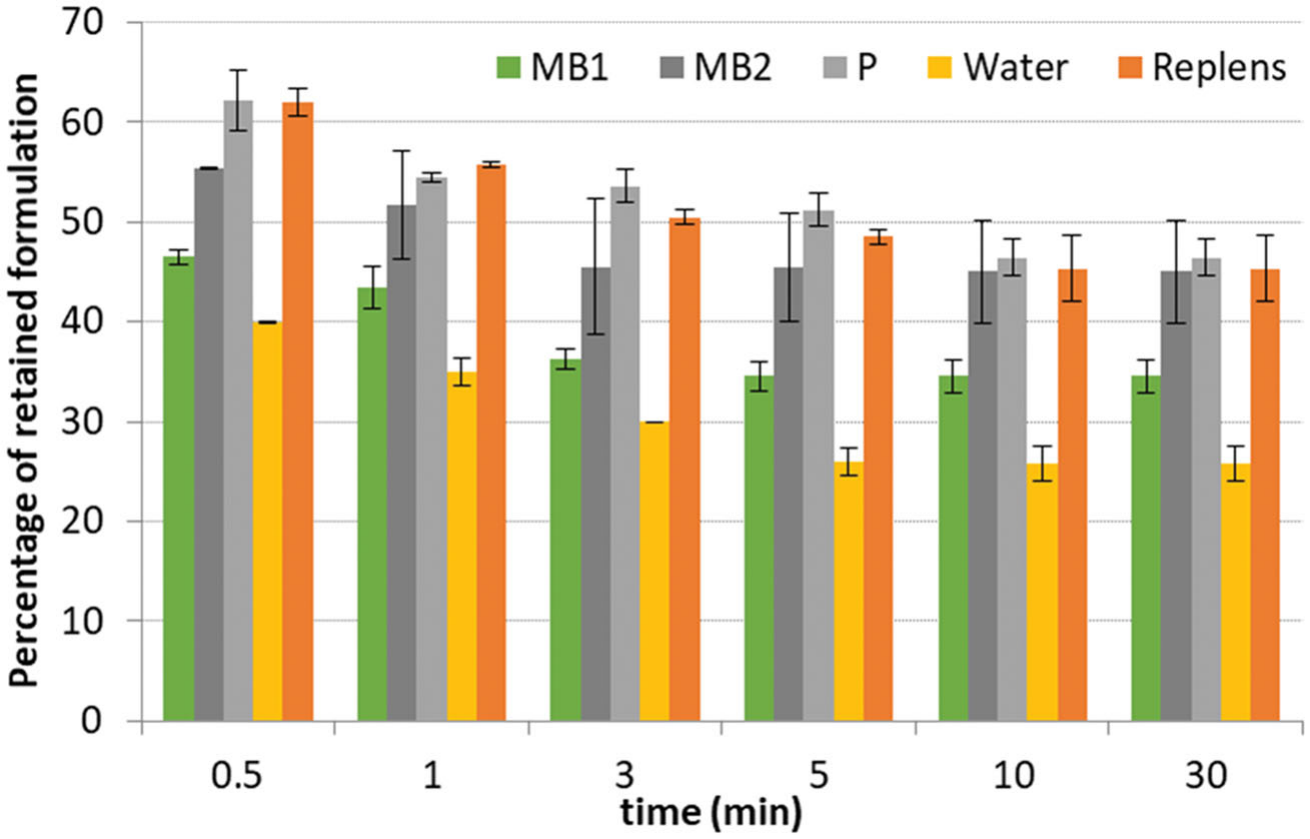
Mucoretention of microparticles MB1–MB2, placebo formulation (P) and controls: (1) water and (2) commercial mucoadhesive vaginal product. Values are expressed as mean ± S.D., *n* = 3.

Microparticles were additionally applied to mucoretention assay to investigate their mucoadhesive characteristic upon dilution with SVF, pH 4.2 (Figure 3).

The assumption of this test was to display whether diluted microparticles’ sample possesses greater ability to retain on the surface of the tissue than the one displayed by water, which interacts only through hygroscopic absorption. Microparticles in contact with SVF formed semi-solid shear-thinning fluids which differed in viscosity values (Table 2). Basically, all formulations flew down gradually from the vaginal tissue segment and the fastest sample slipping was observed within the first minutes of the test. It is worth noting that chitosan microparticles were capable of retaining on the human vaginal epithelium in a similar manner observed for a diluted sample of a commercial vaginal product. The overall detachment from the tissue was approximately 50% of the initial microparticles’ dose in comparison to more than 70% weight loss obtained for water within the first 5 min. MB2 and placebo formulations with greater viscosities were found more resistant to removal from inclined vaginal tissue displaying about 45% retention after 30 min. In turn, MB1 showed less than 35% retention on the tissue surface at the end of the test. Overall, microparticles, even upon dilution with SVF, can stay in contact with the mucosal tissue and assure moderate drug retention on the vaginal epithelium upon mimicking body movements.

### 3.3. Penetration studies

*In vitro/ex vivo* penetration studies are important in evaluating the quality of developed topical formulations in preclinical tests and are considered an essential tool for predicting *in vivo* topical absorption (Frum et al., 2007; Godin & Touitou, 2007; Zhang et al., 2013). Among several approaches (including animal tissues or three-dimensional highly differentiated tissue models, e.g. EpiVaginal^®^) used to evaluate penetration potential, human explants are considered particularly useful in the preclinical stage of microbicide development but, due to limited availability, rarely applied in preclinical studies (Merbah et al., 2011; Patel & Rohan, 2017).

Although chitosan and its derivatives are widely explored as mucoadhesive polymers in the technology of drug carriers for vaginal delivery (Andersen et al., 2015), a limited number of studies have been devoted to the elucidation of the impact of chitosan-based carriers on drug penetration through vaginal tissue (Sandri et al., 2004; Frank et al., 2017). Based on the available literature, chitosan is rather regarded as a penetration enhancer promoting drug absorption either by opening tight junctions or by interacting with extracellular matrix components (Sonaje et al., 2012).

Therefore, in the present studies, we evaluated the penetration behavior of model antiretroviral agent ZVD from gCS microparticles across an excised human vaginal epithelium. Control studies with pure drug suspended in SVF (pH 4.2) with the addition of 3% HEC as a universal gelling agent with no impact on the epithelial transport (Tien et al., 2005) were additionally carried out. The applied model examines the passive diffusion of the active agent across the epithelium solely. To obtain a maximal penetration rate, a drug encapsulated in microparticles was applied on the tissue surface in infinite doses. According to the pKa value (9.68), ZVD was primarily present in a unionized state at SVF (pH 4.2). To fully estimate the influence of a drug carrier on the ZVD ability to permeate across cell membranes, the experimental setup for both control (HEC/SVF formulation) and gCS microparticles assured the maintenance of ZVD in the unionized form.

**Figure 4.**
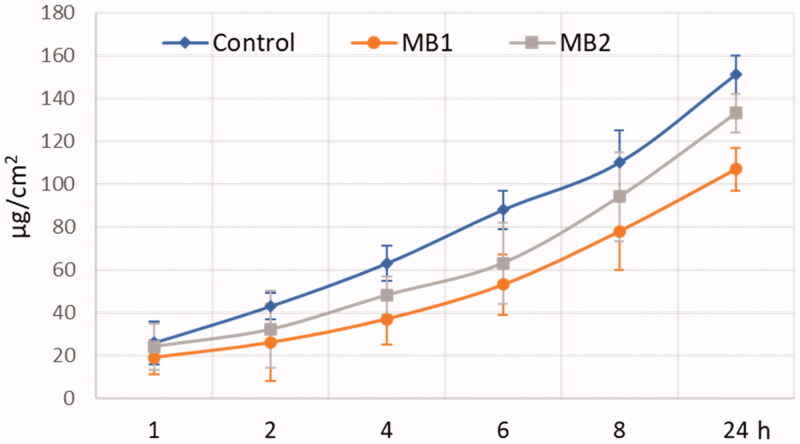
*Ex vivo* permeation (expressed as the amount of agent measured in acceptor medium per tissue area unit) of zidovudine from microparticles MB1, MB2 and control – pure zidovudine in HEC/SVF (pH 4.2 through the human vaginal epithelium). Values are expressed as mean ± S.D. (*n* = 4).

**Figure 5.**
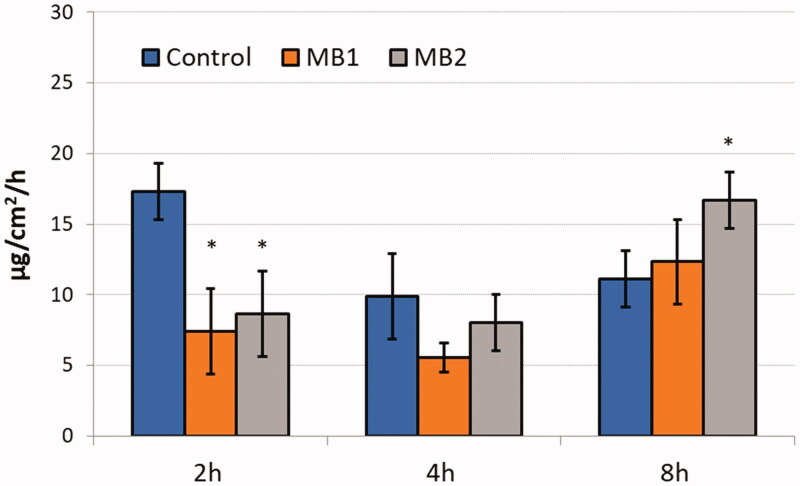
Flux values (expressed as the amount of agent permeated per tissue area unit into the acceptor medium per unit time) of zidovudine from microparticles MB1 and MB2 and control (pure drug dispersed in HEC/SVF, pH 4.2) assessed at 2, 4, and 8 h of studies. *Significant differences with *p* ≤ .05 in comparison to a pure drug in HEC/SVF (pH 4.2). Values are expressed as mean ± S.D. (*n* = 4).

Figures 4 and 5 display the cumulative amount of ZVD permeated over time across the human vaginal epithelium and the permeation flux of the active agent, correspondingly.

In all tests, relatively high ZVD levels were found in the acceptor fluid at an early experimental stage. After 1 h incubation, the permeated fractions of ZVD from microparticles MB1 and MB2 were 18.5 and 23.5 µg/cm^2^. In contrast, in control studies, the amount of active agent in the acceptor medium was above 25 µg per unit tissue’s area. Some differences in penetration behavior were observed between tested formulations and control. Basically, the amount of permeated drug was higher in control studies with ZVD dispersion in HEC/SVF where over 150 µg/cm^2^ of ZVD was present in acceptor medium after 24 h. In contrary, in studies with gCS microparticles, the total amount of ZVD penetrated across excised vaginal tissue after 24 h incubation did not exceed 115 and 135 µg/cm^2^ for MB1 and MB2, respectively (Table 3), suggesting the presence of gCS rather weakened than increased ZVD penetration across the epithelium.

**Table 3.** Quantitative analysis of zidovudine from microparticles MB1 and MB2 and control (pure zidovudine dispersed in HEC/SVF, pH 4.2) in acceptor medium, retained in human vaginal epithelium and on the tissue’s surface (drug recovery from donor compartment) after 24 h incubation (mean ± S.D.; *n* = 4).

		Control		MB1		MB2	
Compartment	Parameter	After 8 h	After 24 h	After 8 h	After 24 h	After 8 h	After 24 h
Acceptor medium	Total amount of permeated drug per tissue’s area (μg/cm^2^)						
		109.9 ± 14.9	150.6 ± 9.2	77.8 ± 17.5	112.3 ± 10.1	96.3 ± 20.3	133.3 ± 9.1
Tissue’s retention^b^	Total amount of drug per tissue’s area (μg/cm^2^)^a^	6.7 ± 0.6		19.6 ± 1.5		13.1 ± 0.7	
Drug recovery from donor compartment^c^	Percent of drug dose (%)	94.0 ± 1.9		95.1 ± 2.2		91.2 ± 1.0	

^a^ Dispersion of microparticles-loaded ZVD (MB1–MB2) or ZVD in HEC/SVF (which amount corresponded to dose 5 mg) was placed on the tissue surface with an area of 0.81 cm^2^; concentrations of ZVD were determined in: ^b^solvent used for tissue extraction and ^c^drug dispersion aspirated from the surface of the tissue.

This behavior is rather surprising, as chitosan has been considered so far as a penetration enhancer that facilitates drug absorption through mucosal membranes (Sandri et al., 2004; Caramella et al., 2010). For instance, Frank et al. (2014) demonstrated that chitosan nanocapsules were able to increase the Nile red penetration through vaginal tissue (Frank et al., 2014), whereas studies on formulation based on chitosan gel revealed that the presence of chitosan led to a higher permeation degree of imiquimod into vaginal tissue (Frank et al., 2017). It is worth noting that lower flux values of ZVD from microparticles were observed in the first hours of studies as compared to control examination with ZVD dispersion in HEC/SVF (Figure 5). This appeared desirable since antiretroviral reverse transcriptase inhibitors (such as ZVD) are related to a number of systemic adverse reactions (Sweetman, 2011). The presence of a higher amount of gCS in MB2 (with gCS:ZVD mass ratio 5:1) was responsible for a slight increase in the drug flux at 8 h of analysis, which may be beneficial when it comes to assuring prolonged microbicide activity.

**Figure 6.**
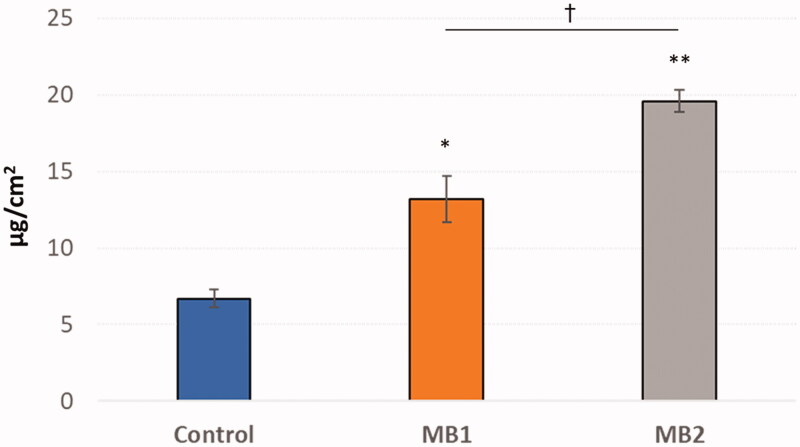
*Ex vivo* retention (expressed as the amount of agent accumulated per tissue area unit) of zidovudine from microparticles MB1 and MB2 or control (pure zidovudine in HEC/SVF, pH 4.2) in human vaginal epithelium measured after 24 h (mean ± S.D., *n* = 4). *^,^**Significant differences with *p* ≤ .05 in comparison to control – pure drug in HEC/SVF (pH 4.2); ^†^substantial differences with *p* ≤ .05 between microparticles MB1 and MB2.

The amount of drug recovered from the donor compartment after 24 h incubation (Table 3) fulfilled the criteria of acceptation (according to EMA, the acceptable limit of overall recovery is 90–100%) and confirmed the accuracy of the applied research method. Figure 6 presents the retention of ZVD in human vaginal epithelium after 24 h.

The degree of drug accumulation in studies using MB was relatively high and varied between 13.8 µg/cm^2^ and 19.6 µg/cm^2^ for MB1 and MB2, respectively. It is worth noting that the presence of gCS was found to improve the tissue retention of model microbicide agent as the amount of drug retained in the vaginal epithelium was significantly greater for microparticles MB1–MB2 as compared to control. A significantly higher ZVD tendency to tissue accumulation was observed in the studies using MB2, in which a faster rate of drug permeation was simultaneously observed. Therefore, it may be assumed that formulation MB2 potentially better comply with the requirements for microbicide drug delivery systems, and relatively high microbicide retention in epithelium may assure protective activity against viral transmission.

### 3.4. *In vitro* anti-HSV 2 activity

Genital herpes is one of the most common ulcerative STDs, particularly in developing countries. Currently, anti-HSV treatment is based on symptomatic therapy with nucleoside analogs inhibiting viral replication (e.g. acyclovir). Despite acyclovir and its analogs are regarded as first-line drugs with a high safety profile, viral resistance is still an important limitation of effective treatment (Gupta et al., 2007; Sauerbrei et al., 2010).

Among STDs therapeutic strategies (including HSV infections), the development of topically (vaginally, rectally) applied microbicides is regarded as an attractive approach in preventing mucosal transmission of pathogens. From microbicide agent, it is expected that it will bind cell-free virus with high affinity and efficiently block its further spread to uninfected cells. Simultaneously, an ideal microbicide delivery system should provide intimate contact with the mucosal tissue and protect from infection by the residual active virus.

Despite chitosan and its derivatives are considered as polymers with antiviral potential capable of suppressing viral infections, the mechanism of its activity toward the HSV virus is poorly understood. In the present study, we hypothesized that chitosan impacts the direct contact of the herpes virus with mucosal cells. Thus, the goal was to assess the antiviral effect of gCS microparticles toward HSV-2 infection using viral attachment and penetration assay. The tests were performed in two keratinocyte cell lines commonly used to study microbicides *in vitro* (Michaelis et al., 2019; He et al., 2020). To test whether viral binding to the cell surface can be affected, HaCaT and VK2-E6/E7 cells were initially incubated at 4.0 ± 1 °C with HSV-2 in the presence of gCS microparticles, then the temperature was raised to 37.0 ± 1 °C to allow the virus to enter the cells. In the penetration assay, HSV-2 was initially allowed to attach to the cell surface at 4.0 ± 1 °C, then the unbound virus was removed, and gCS microparticles were applied at 37.0 ± 1 °C to test if formulations could block the virus from entering the cells. Based on the results from mucoadhesive and penetration studies, microparticles MB2 with polymer to drug mass ratio 5:1 and corresponding placebo formulation were chosen for *in vitro* anti-HSV tests at a concentration previously found nontoxic to the host cells (Szymanska et al., 2019).

**Figure 7.**
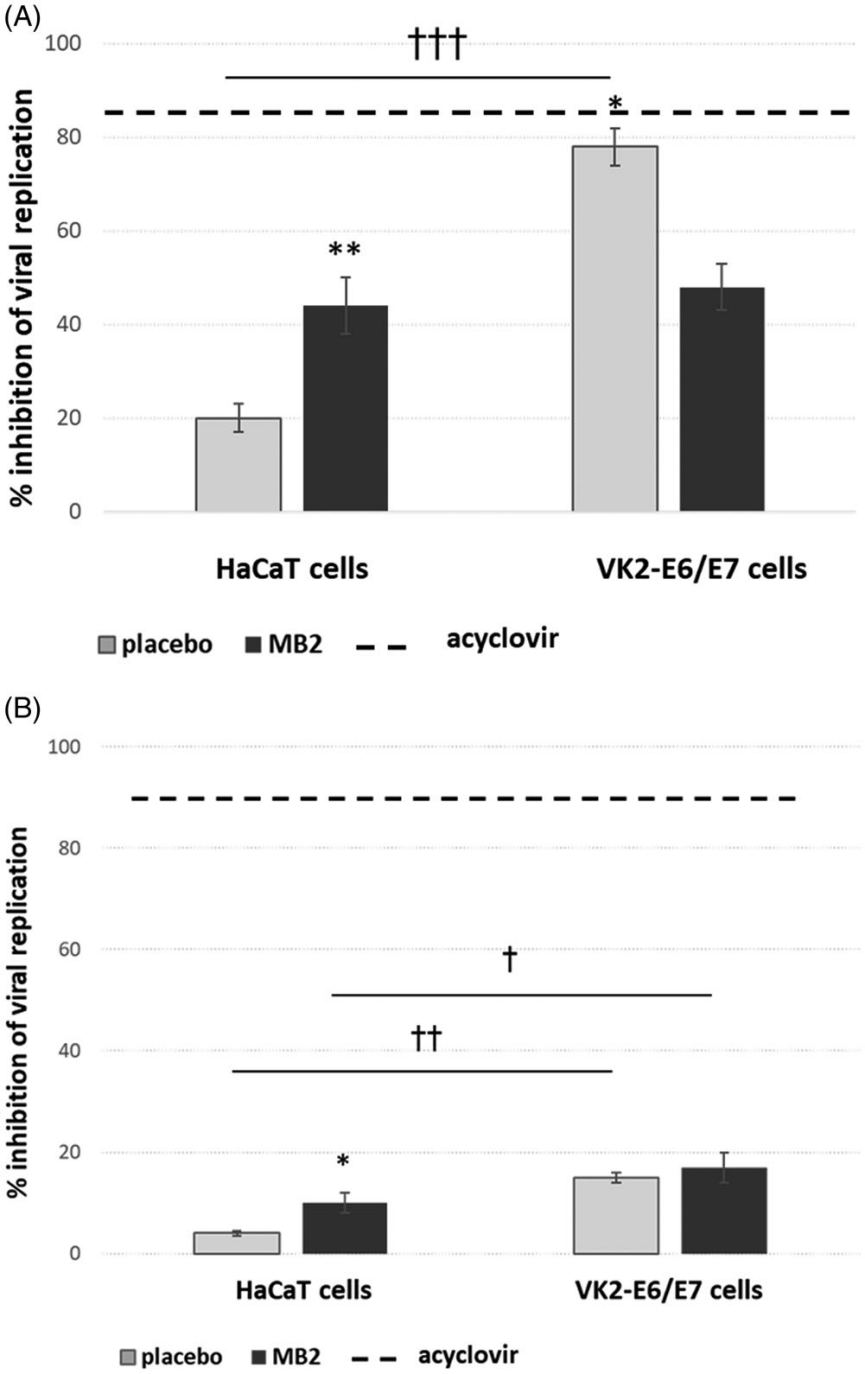
(A) HSV-2 attachment inhibition and (B) HSV-2 penetration inhibition (expressed in %) in HaCaT and VK2-E6/E7 cells in the presence of microparticles MB2 and the corresponding placebo formulation as compared to control acyclovir. At 24 hours post infection cells and supernatants were collected and titrated to determine gB copies per ng DNA compared to untreated HSV-2 infected cultures (mean ± S.D.); *significant differences with *p* ≤ .05, and **p ≤ .01, in comparison to the placebo, while ^†^substantial differences with *p* ≤ .05, ^††^significant differences with *p* ≤ .01 and ^†††^*p* ≤ .001, respectively between HaCaT and VK2-E6/E7.

The results presented in Figure 7(A) displayed the ability of gCS microparticles to affect HSV-2 attachment. However, profound differences in formulations’ activity toward HSV-2 between standard cell line HaCaT and vaginal epithelial cells were noted. Placebo formulation was found to be more effective in preventing viral attachment, especially regarding VK2-E6/E7 infected cells. Incubation with drug-free microparticles was responsible for approximately 80% inhibition of HSV-2 binding to the VK2-E6/E7 cell surface after 24 h post infection. In turn, MB2 was more effective in HaCaT cells, and the rate of reduction in HSV-2 titers was about 50%. It is worth noting that placebo gCS microparticles blocked virus attachment in a similar manner as control acyclovir in vaginal epithelial cells. The observed alterations in the susceptibility of HSV-2 to tested formulations between cell lines might be related to different characteristics of the cell types. VK/E6E7 cells are vaginal epithelial cells, so they much better imitate *in vitro* conditions observed in tests with the vaginal epithelium.

The results of the penetration assay displayed that HSV-2 was not significantly impaired (*p* > .05) for entry into HaCaT cells following exposure to both placebo or MB2 microparticles (Figure 7(B)). When tested in VK2-E6/E7 cells, both tested formulations inhibited viral entry comparably and showed less than 20% decrease in titers after 24 h incubation. These results of anti-HSV-2 inhibition are considerably lower than those received in the attachment assay.

Based on obtained data, it can be assumed that gCS itself exhibits anti-HSV-2 activity *in vitro*. It is known that HSV entry and transmission require a combination of viral surface glycoproteins, including gD, gB, and gH-gL heterodimer (Chowdhury et al., 2012). With regard to the polycationic nature of chitosan and its ability to interact with glycosaminoglycans and glycoproteins present in the extracellular matrix, gCS may impair the function of these structural molecules engaged in HSV transmission. The plausible mode of gCS action involved in HSV-2 inactivation could be related to interaction with the virus surface through reactive amino moieties and the creation of a physical barrier that prevents viral interaction with the host cells receptors.

## 4. Conclusions

The obtained findings point to the feasible mucoadhesive and mucoretention behavior of gCS microparticles in contact with isolated human vaginal epithelium, which ability to adhere to mucosal tissue was comparable to those attained for the commercial mucoadhesive vaginal product. The *ex vivo* studies on passive diffusion of drug through the vaginal tissue revealed that gCS increases the accumulation of model microbicide in the vaginal epithelium but surprisingly does not facilitate its penetration across human vaginal tissue. It is worth noting that penetration and retention characteristics of model microbicide may be modulated by alteration of the polymer–drug mass ratio in microparticles’ formulation. These results also demonstrated that gCS itself exhibits anti-HSV-2 activity *in vitro*, and the mode of action is related to blocking viral attachment. Overall, presented findings indicate that gCS microparticles comply with the requirements for microbicide drug carriers and hold promise in STDs prophylactic strategy as antiherpes adjunctive platforms. However, this assumption requires further *in vivo* studies to assess the examined carriers' efficacy and safety profile.
